# Supplementary figures and images for: Atmospheric particulate matter aggravates CNS demyelination through involvement of TLR-4/NF-kB signaling and microglial activation
Source: eLife. 2022 Feb 24;11:e72247. doi: 10.7554/eLife.72247 (PMC8893720; doi:10.7554/eLife.72247)

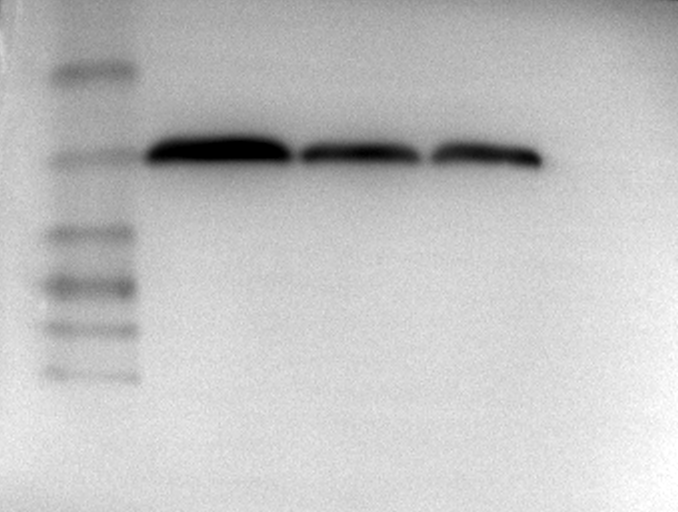

Supplement: Source data 1. [file elife-72247-data1.zip › Source Data/Figure 4 -Source Data 2 Raw source data for G.tif]

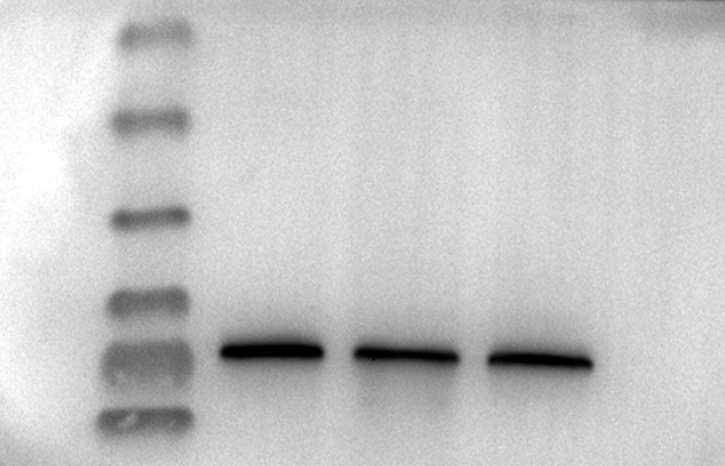

Supplement: Source data 1. [file elife-72247-data1.zip › Source Data/Figure 4 -Source Data 3 Raw source data for G.tif]
